# Brain structural parameters correlate with University Selection Test outcomes in Chilean high school graduates

**DOI:** 10.1038/s41598-022-24958-0

**Published:** 2022-11-29

**Authors:** Daniza Ivanovic, Francisco Zamorano, Patricia Soto-Icaza, Tatiana Rojas, Cristián Larraín, Claudio Silva, Atilio Almagià, Claudia Bustamante, Violeta Arancibia, Francisca Villagrán, Rodrigo Valenzuela, Cynthia Barrera, Pablo Billeke

**Affiliations:** 1 Laboratory of Nutrition and Neurological Sciences, Human Nutrition Area, Institute of Nutrition and Food Technology Dr. Fernando Monckeberg Barros (INTA), University of Chile, Santiago, Chile; 2 Laboratorio de Neurociencia Social y Neuromodulación, Centro de Investigación en Complejidad Social (neuroCICS), Facultad de Gobierno, Universidad del Desarrollo, Santiago, Chile; 3 Unidad de Imágenes Cuantitativas Avanzadas, Departamento de Imágenes, Clínica Alemana, Universidad del Desarrollo, Santiago, Chile; 4 Radiology Department, Facultad de Medicina-Clínica Alemana, Universidad del Desarrollo, Santiago, Chile; 5 Laboratory of Physical Anthropology and Human Anatomy, Institute of Biology, Faculty of Sciences, Pontificia Universidad Católica de Valparaíso, Valparaíso, Chile; 6 Department of Global Partnership for Education (GPE) World Bank, Washington, USA; 7 Department of Nutrition, Faculty of Medicine, University of Chile, Santiago, Chile

**Keywords:** Neuroscience, Neurology

## Abstract

How well students learn and perform in academic contexts is a focus of interest for the students, their families, and the entire educational system. Although evidence has shown that several neurobiological factors are involved in scholastic achievement (SA), specific brain measures associated with academic outcomes and whether such associations are independent of other factors remain unclear. This study attempts to identify the relationship between brain structural parameters, and the Chilean national University Selection Test (PSU) results in high school graduates within a multidimensional approach that considers socio-economic, intellectual, nutritional, and demographic variables. To this end, the brain morphology of a sample of 102 students who took the PSU test was estimated using Magnetic Resonance Imaging. Anthropometric parameters, intellectual ability (IA), and socioeconomic status (SES) were also measured. The results revealed that, independently of sex, IA, gray matter volume, right inferior frontal gyrus thickness, and SES were significantly associated with SA. These findings highlight the role of nutrition, health, and socioeconomic variables in academic success.

## Introduction

The learning process is a multidimensional issue that depends on several elements related to the child, the families, and the educational system^1–3^. Studies about scholastic achievement (SA) have shown that several neurobiological factors impact academic performance. Nonetheless, specific brain measures that independently influence the SA have not been sufficiently investigated in school-age students. This fact is especially relevant in the analysis of the University Selection Test (PSU, Prueba de Selección Universitaria), the national baccalaureate examination for admission to Chilean universities, which has obvious implications for the future of the students as a result of ranking by score.

The intellectual ability (IA)^4^ is the most studied and relevant factor that impacts SA^5–7^. Several studies have highlighted the association between IA and brain structures^8–12^. Most research indicates that gray matter volume (GMV), rather than white matter (WM), correlates with IA^12^. During a child’s development, brain volume and head circumference (HC) also positively correlate with IA^13,14^. Other findings distinguish differential contributions of GMV and WM microstructure connections to individual differences in intelligence and memory, respectively^15^. While GMV correlates with IA^8,9,12^, the relationship between GMV and specific cognitive abilities is not straightforward. For example, studying a large Magnetic Resonance Imaging (MRI) sample of school-age students, research has found only a significant correlation between GMV and single-word reading in adolescents separated by sex^16^. Thus, the weight of specific cognitive function and whole brain functioning in the relationship between GMV and IA is unclear.

Despite the preceding findings, the neurobiological factor underlying SA has just begun to be studied. The evidence has noted that HC and brain size correlate with SA^17–20^. Our prior research also indicated that broad brain volume measures correlate with SA^5^. A recent study shows that cortical thickness can accurately classify individuals with high and low SA^21^. Furthermore, functional connectivity of some brain areas, including the inferior frontal cortex, correlates with SA^22^. However, when IA is considered in statistical modeling, the broad brain measure loses an association with SA. The preceding research data demonstrates that more precise measurements of brain morphometry are needed in order to affirm an association with SA.

Another relevant factor that impacts SA is an early averse social environment, which disturbs brain maturation with potential implications for mental health^23^. For instance, malnutrition alters HC, brain development, and intelligence; poverty and deprivation exacerbate these adverse effects that persist at least into childhood and adolescence^24–31^. Thus, early postnatal nutrition is essential for brain growth and maturation, impacting WM connectivity and long-term cognitive functions^32,33^. Several authors have emphasized the importance of particular omega-3 polyunsaturated fatty acid patterns on SA, IA, and brain structural volumes^34–36^. Along this same line of research, low socioeconomic status (SES) and the experience of traumatic, stressful events impact brain development^37,38^. Accordingly, both early life and current SES significantly help to explain the variance of gray matter^39,40^.

Overall, the evidence suggests that brain volume is associated with SA. Nonetheless, the role of specific brain areas in this relationship is unclear, as is whether this association is independent of other factors, such as IA or nutritional status. Hence, this study aimed to relate the brain structural parameters and the results of the PSU in Chilean high school graduates within the framework of a multidimensional approach considering socio-economic, intellectual, nutritional, and demographic variables. The purpose was to test the hypotheses that (i) brain parameters such as GMV independently correlate with PSU scores and that (ii) IA, GMV, SES, and sex are the most relevant parameters that explain PSU outcomes variance.

## Methods

### Design

This is an observational, cross-sectional, and comparative study.

### Description of the population

The target population, 96,197 students (39% of the Chilean school population), included all school-age participants enrolled in the first grade of high school (HSG) in the urban area of the Metropolitan Region of Chile in 2010 (Chile, Ministerio de Educación, 2009). They belonged to the public, private-subsidized, and private non-subsidized schools and were distributed in 1,262 educational establishments, as was described in previous studies^1,2^.

### Description of the sample

**Figure 1:**
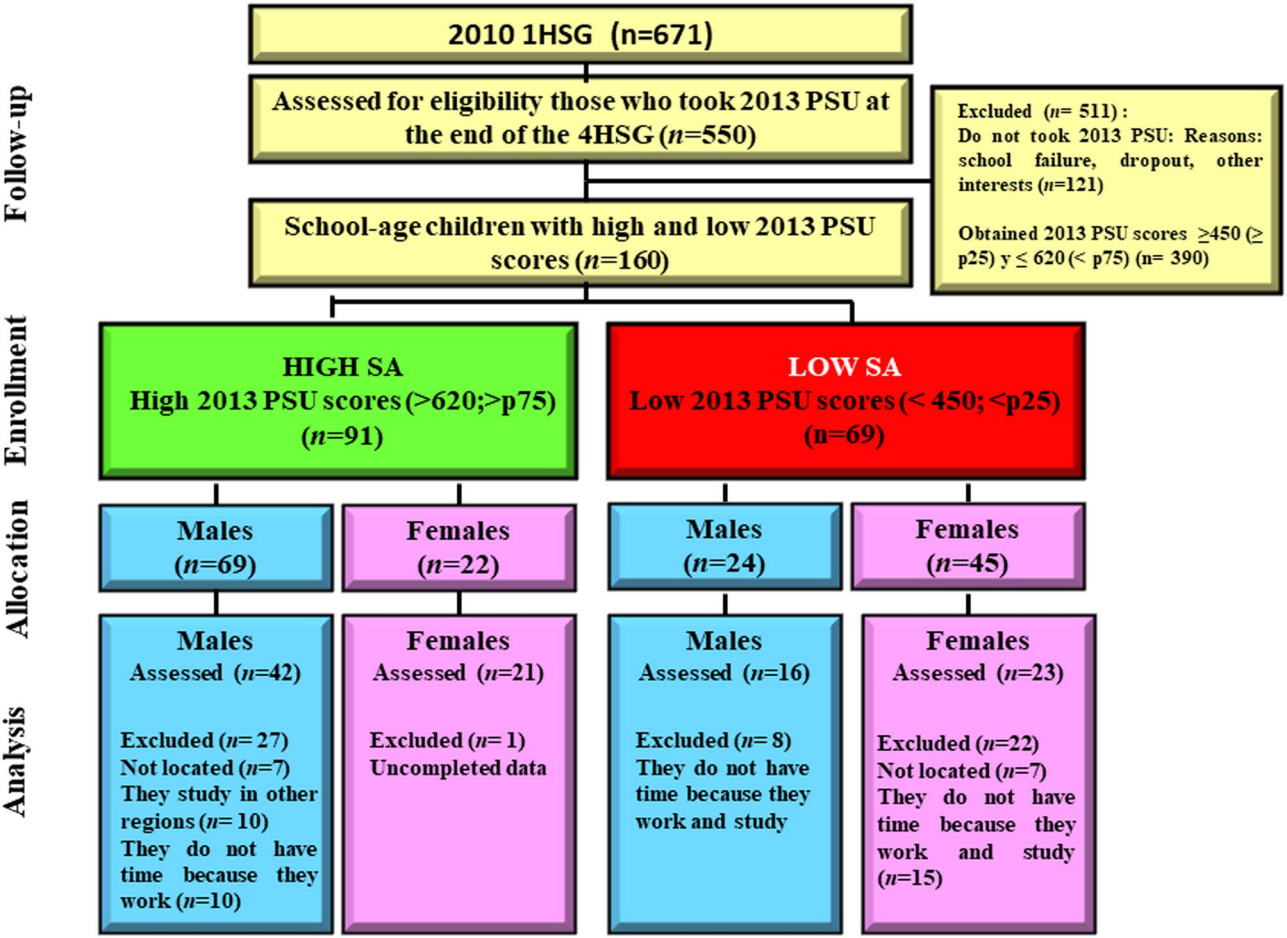
Flow diagram of the sample selection and distribution of the sample by group and sex. PSU: University Selection Test; 1HSG: the first high school grade; 4HSG: the fourth high school grade; SA: scholastic achievement.

The sampling plan was widely described in our previous studies^1,2^. A representative sample of 671 school-age students of the 2010 first HSG and their parents, the school principals, and teachers agreed to participate and signed the informed consent form. At the end of 2013, the students of the 2010 first HSG graduated from the fourth HSG and took the PSU. A total of 550 and 548 school-age participants took the language and mathematics PSU tests, respectively. All the school-age students (*n* = 160) who obtained high (*n* = 91) or low PSU scores (*n* = 69) in both language and mathematics were invited to participate in the study. A high PSU score was defined as greater than 620 in both tests, representing the 75th percentile at both the sample and national levels. In contrast, a low PSU score was defined as less than 450 in both tests, representing the 25th percentile at both the sample and national levels. Note that the PSU score is a normalized scale with a mean of 500, a standard deviation of 110, a minimum score of 150, and a maximum of 850. A total of 102 healthy high school graduate students born at term voluntarily agreed to participate and signed the informed consent form. All of them were successfully scanned by MRI. All participants had no history of alcoholism, neuropsychiatric diagnosis, symptoms of brain damage, intrapartum fetal asphyxia, hyperbilirubinemia, epilepsy, or heart disease, and their mothers had no history of smoking, alcoholism, or drug intake before and during pregnancy. Participants' age ranged from 17.3 y to 20.3 y (mean age = 18.2 ± 0.5 y). In the High SA Group, 75.8% of the high PSU scores were obtained by males, and in the Low SA Group, 65.2% of the low PSU scores were obtained by females (*p* < 0.0001). Figure 1 shows the flow diagram of the sample selection and distribution by group and sex.

### Brain structural parameters: data acquisition

Images were acquired at the Radiology Department of the Clínica Alemana de Santiago with a 3 T Siemens Skyra scanner and a 20-channel head coil. Participants were prepared for the MRI and were instructed to relax and keep still during image acquisition. For each subject, a 3D structural T1-weighted scan [voxel size, 1 × 1 × 1 mm; slices per slab, 176; field of view (FoV), 256 mm; repetition time (TR) = 2.53 s; echo time (TE) = 2.19 ms]. Cortical and subcortical segmentation and cortical thickness were obtained using FreeSurfer 6 (http://surfer.nmr.mgh.harvard.edu) methods using volumetric T1 imaging^41–43^. Cortical thickness, defined as the shortest distance between the gray-white matter boundary and the outer cortical boundary, was measured at each vertex across the surface. Cortical thickness surface maps were smoothed with a Gaussian kernel of full width at a half-maximum of 10 mm.

### SES

SES was measured using the Graffar Modified Scale adapted for Chilean urban and rural populations, which considers the following socioeconomic indicators: parental schooling and occupation of the household head and the housing characteristics (building materials, ownership, water supply, and ownership of durable goods)^44^. Specifically, the data of SES was obtained through an interview with the student's mother. A six-point scale was obtained as follows: High SES = 1, 2 points; Middle SES = 3 points; Low SES = 4, 5 points; Extreme poverty SES = 6 points.

### IA

IA was assessed with the standard version of the Raven's Progressive Matrices Test (RPMT) in book form, with a general scale for children of 12 years and above that had been standardized for Chilean school-age students^19,45^. The Standard RPMT is a non-verbal test and, in any of its forms, constitutes one of the tests most frequently applied for quantification of general intelligence, evidencing a robust and reliable measure of the general intelligence factor^46^. The test was administered collectively in the classrooms by an educational psychologist. WHO experts for developing countries have recommended applying Raven’s test because its results are not affected by culture^31^. Scores were registered as a percentile scale according to age, in the following grading: Grade I = Superior Intellectual Ability (score ≥ p95); Grade II = Above Average (score ≥ p75 and < p95); Grade III = Average (score > p25 and < p75); Grade IV = Below Average (score > p5 and ≤ p25) and Grade V = Intellectually Deficient (score ≤ p5). For the analyses, IA grades were pooled into two groups: I + II and III + IV + V. The rationale aimed to obtain two groups of participants as equitably and balanced as possible to estimate statistical parameters, that is, to obtain no more than the 20% of the cells having smaller amounts than 5.

### Nutritional status

The prenatal nutritional background and early nutritional measurements, such as birth weight, body length, and duration of breastfeeding, were registered. Measurements of weight (W), height (H), and head circumference (HC) were carried out at school using standardized procedures (Gibson, 1990). The postnatal nutritional background was expressed as height-for-age Z-score (Z-H) according to NCHS-CDC tables^47^. The head circumference-for-age Z-score (Z-HC) was assessed using tables^48–50^. Z-HC values were similar when applying these tables (the correlation coefficient between these patterns was 0.98). Finally, Z-HC values were calculated using the tables of Roche et al.^50^. The current nutritional status was expressed as body mass index (BMI, weight/height^2^, NCHS-CDC tables^48,49^). BMI values are commonly categorized as follows: underweight (BMI < 18.5), healthy weight (BMI between 18.5 to < 25), overweight (BMI is 25.0 to < 30), and obesity (BMI is 30.0 or high), although in the present study values were expressed as mean ± SD. Higher BMI values are related to a high proportion of body fat and, as a result, poor nutritional status. Note that, in the current sample, only 2 participants had BMI < 18.5. BMI was calculated using biological age derived from the Tanner stages. Birth weight and birth length were used as indices of prenatal nutrition, Z-H and Z-HC served as indicators of postnatal nutritional background, and Z-BMI was used as an index of current nutritional status.

### PSU

Results from the PSU outcomes in language and mathematics tests were registered for the 2010 first HSG school-age students when they graduated from the fourth HSG in 2013. PSU has a maximum score of 850 and a minimum of 150 for each test (language and mathematics tests with 80 items each) and was expressed as mean ± SD. Scores below 450 bar students from applying to universities. PSU was considered a dependent variable.

### Statistical analysis

Data were analyzed using analysis of variance (ANOVA) and t-test for comparison of means after applying the Shapiro–Wilk test to establish whether the distribution of the variables was normal. Multiple comparisons were corrected by Bonferroni's test. Non-parametric tests (chi-square) were used for categorical variables. Pearson correlation coefficient was used to establish interrelationships between variables. Partial correlations were used to control for the interdependence of different brain volumes within subjects, as has been proposed and used for structural brain data^51–53^. The correlation values with brain structural parameters were corrected by the false discovery rate (FDR, q < 0.05). The determination coefficient (R^2^) was calculated to measure the fit of the regression models. Pearson and Spearman correlation coefficients were used for quantitative and ordinal variables, respectively. The stepwise procedure was used in the linear regression analysis to establish the most important independent variables affecting PSU (mean language + mathematics), language, and mathematics scores (dependent variable). The brain parameter initially evaluated for the selection method involved all those structures with PSU (language or mathematics) correlation greater than 0.5 (abs(r) > 0.5). For all hypothesis tests, the level of significance was < 0.05 two-tails. All the comparisons were carried out separately by sex, except when sex was included as an independent regressor (Whole-brain analysis and general linear model) and in the PSU score in the demographic descriptions (Table 1). Note that in the preceding case, the between-group comparison does not have relevance because the PSU score was the selection criteria for the group selection.

**Table 1:** Mean age, University Selection Test scores, socioeconomic and intellectual variables by group and sex.

Variables	Males			Females			Within group comparison	
	High SA Group^#^ (n = 42)	Low SA Group^#^ (n = 16)	*p-*value	High SA Group^#^ (n = 21)	Low SA Group^#^ (n = 23)	*p-*value	High SA Group^#^ *p*-value	Low SA Group^#^ *p*-value
**Demographic**								
Age (y)	18.1 ± 0.3	18.6 ± 0.8	.0003***	18.0 ± 0.3	18.1 ± 0.5	.3582	.4795	.0292*
**University Selection Test (PSU) scores**								
PSU (L + M)	712 ± 41	395 ± 56		672 ± 42	388 ± 55		.0005***	.7209
Language (L)	690 ± 50	403 ± 72		665 ± 53	388 ± 73		.0761	.5317
Mathematics (M)	734 ± 61	390 ± 83		677 ± 69	388 ± 68		.0013**	.9392
**Socioeconomic status (SES)**								
SES (high + medium)	81.0	56.2		84.2	13.0		X_o_^2^ = 34.0038 df = 3 *p* < .0001***	
SES (low)	19.0	43.6		15.8	87.0			
**Intellectual ability (IA)**								
IA (I + II)	97.6	18.8		90.5	4.4		X_o_^2^ = 75.5633 df = 3 *p* < .0001***	
IA (III)	2.4	56.2		9.5	60.8			
IA (IV + V)	0.0	25.0		0.0	34.8			

Data are expressed as mean ± SD, Means were compared by Bonferroni's test;

Data for SES are expressed in percentage of cases for SES categories and compared by Chi-square test.

Data for IA are expressed in percentage of cases for IA categories and compared by Chi-square test. IA grades were pooled in two groups: I + II, and III + IV + V. IA grades: Grade I, superior; Grade II, above average; Grade III, average; Grade IV, below average; Grade V, intellectually deficient. p < .05 *; < .01**; < .001 ***

^#^ SA: scholastic achievement; High SA Group: High PSU score (> 620, > p75); Low SA Group: Low PSU score (< 450, < p25).

Data were processed using the Statistical Analysis System package (SAS 9.3, SAS Institute Inc. (Cary, NC). Whole-brain analyses across the cortical surface vertex were performed to reduce the risk of Type II errors. These analyses were carried out using Surfstat (http://www.math.mcgill.ca/keith/surfstat/), a toolbox created for MATLAB (The MathWorks, Inc., Nathan, MA). Random field theory (RFT) corrections (cluster corrected *p* < 0.05, cluster threshold detection, CTD, p < 0.001) were used to account for multiple comparisons^54^. In order to incorporate the results from the whole-brain analyses of the cortical surface, the right inferior frontal gyrus and the left inferior frontal gyrus (for completeness) volumes were extracted using an independent ROI from the area A45c_r and A45c_l of the Brainnetome atlas (https://atlas.brainnetome.org/)^55^.

### Ethical approval and consent to participate

The experimental protocol and all methods were performed in accordance with institutional guidelines and were approved by the Ethics Committee in Studies in Humans of the Institute of Nutrition and Food Technology Dr. Fernando Monckeberg Barros (INTA), University of Chile, and ratified by the Bioethics Committee of the National Fund for Scientific and Technological Development (FONDECYT), Chile. The participants' informed consent was obtained according to the norms for Human Experimentation, Code of Ethics of the World Medical Association (Declaration of Helsinki).

## Results

### Sample

From a representative cohort of school-age students^1,2,35^, a sample of 102 participants was successfully scanned after they completed the University Selection Test (PSU). This sample represents 69% and 56% of students who obtained the highest (High SA Group) or the lowest (Low SA Group) PSU scores, respectively (see Methods for further details). The demographic descriptions of the sample are shown in Table 1. Menarcheal age did not differ significantly between females in High SA (12.6 ± 1.2) and Low SA groups (12.5 ± 1.2) (*F* = 0.12; *p* = 0.7359). Males from the High SA Group obtained higher scores in the PSU than females from the same group (*p* = 0.0005), which is explained by their higher scores in mathematics (*p* = 0.0013).

### Comparison of family SES

A significant difference among SES categories by sex and group was found (*X*_*o*_^2^ = 34.0038; df = 3; *p* < 0.0001). In the High SA Group, most participants, 81% and 84.2% of males and females, respectively, belonged to the high and medium SES. While in the Low SA Group, the percentage of participants that belonged to the high and medium SES was 56.2% and 13% for males and females, respectively. To note, most females of the Low SA Group belonged to low SES (87%) (Table 1). The parents and the head of the household of the school-age children from the High SA Group had higher levels of schooling and jobs and lived in better housing quality than their peers with low SA (*p* < 0.0001). SES, as well as socioeconomic indicators (schooling and occupation of the household head and the housing characteristics), were positively and significantly associated with PSU outcomes both in the language and mathematics tests (*p* < 0.0001).

### IA

IA was estimated through Raven's Progressive Matrices Test (see "Methods"). A significant difference among AI categories by sex and group was found (*X*_*o*_^2^ = 75.5633; df = 3; *p* < 0.0001; detailed results are shown in Table 1). To note, a great percentage of participants of the High SA Group exhibit an IA in the categories I + II (97.6% and 90.5% for males and females, respectively). However, in the Low SA Group, males and females registered mainly IA grade III, followed by grades IV + V.

### Prenatal, postnatal, and current nutritional status

Table 2 shows that birth weight and birth height values were significantly lower in males from the Low SA Group than in males from The High SA Group (*p* = 0.0135 and *p* = 0.0175, respectively). Z-HC was lower for females from the Low SA Group than in the High SA Group (*p* = 0.018). Although in the High SA Group and the Low SA Group, the means of BMI in males corresponded to the current nutritional status of healthy weight, and in females to overweight, BMI values did not show significant differences between the groups.

**Table 2:** Prenatal, postnatal, and current nutritional status by group and sex.

Nutritional indicators	Males			Females		
	High SA Group (n = 42)	Low SA Group (n = 16)	*p-*value	High SA Group (n = 21)	Low SA Group (n = 23)	*p-*value
**Prenatal nutritional background**						
Birth weight (g)	3559 ± 591	2987 ± 717	.0135*	3236 ± 441	3399 ± 557	.3577
Birth height (cm)	50.8 ± 2.8	48.3 ± 3.1	.0175*	49.8 ± 2.8	49.6 ± 1.8	.8470
**Postnatal nutritional background**						
Z-HC	0.80 ± 1.03	0.44 ± 1.13	.2491	0.16 ± 1.03	- 0.63 ± 1.09	.0182*
**Current nutritional status**						
BMI	23.4 ± 3.2	25.3 ± 5.3	.0991	24.0 ± 3.5	25.7 ± 5.1	.2206

Results are expressed as mean ± SD. Means were compared by Bonferroni's test. SA: scholastic achievement; PSU: University Selection Test; High SA Group: High PSU scores (> 620, > p75); Low SA Group: Low PSU scores (< 450, < p25). Z-HC, head circumference-for-age Z-score; BMI: body mass index. p < .05 *; < .01**; < .001 ***

### Brain structural parameters volumes

**Figure 2:**
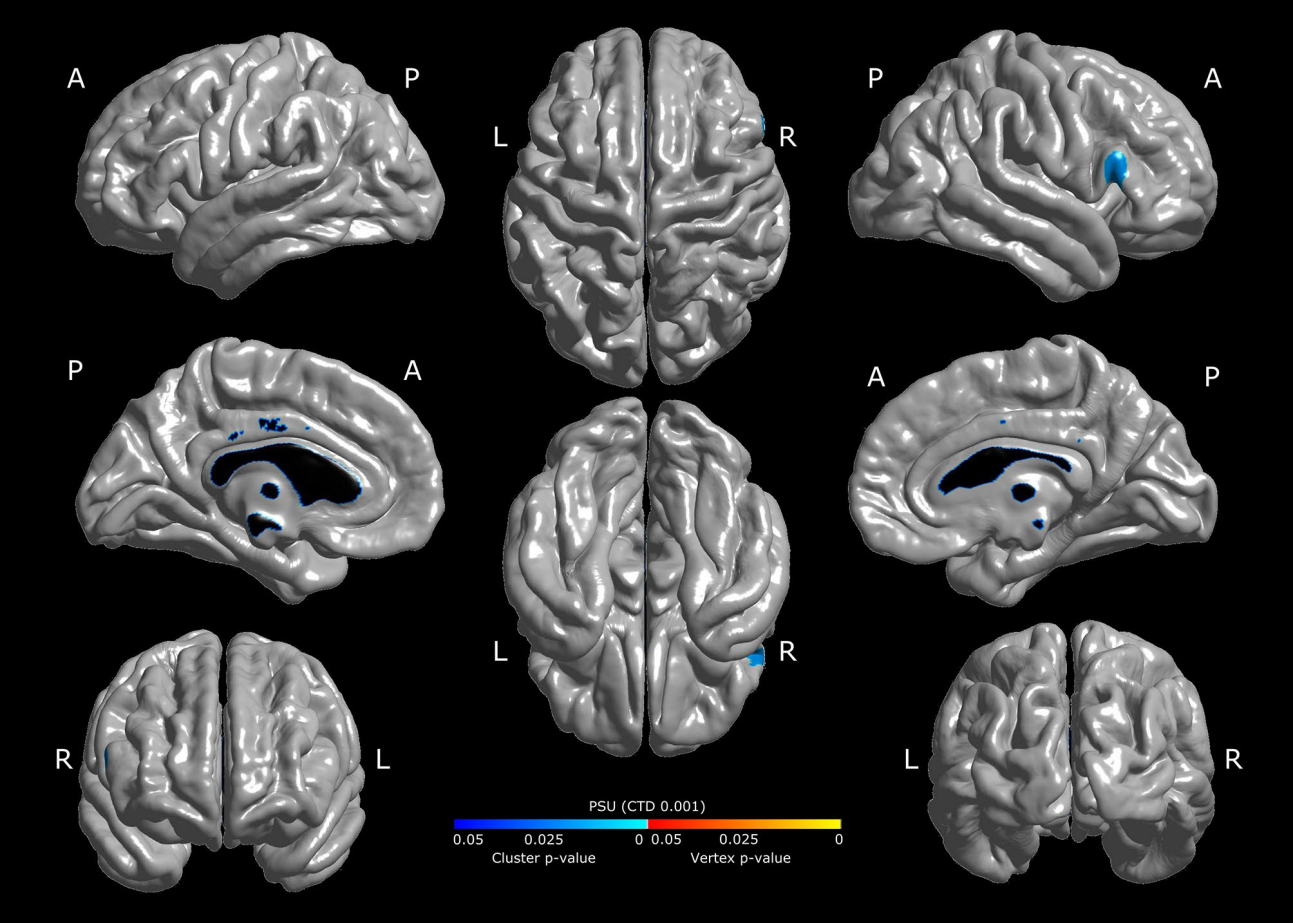
Cortical thickness results and their correlation with the University Selection Test outcomes (PSU) (language + mathematics), corrected by sex and socioeconomic status. Colors represent the T-Value per vertex. A: anterior; P: posterior, R: right, L: left.

**Figure 3:**
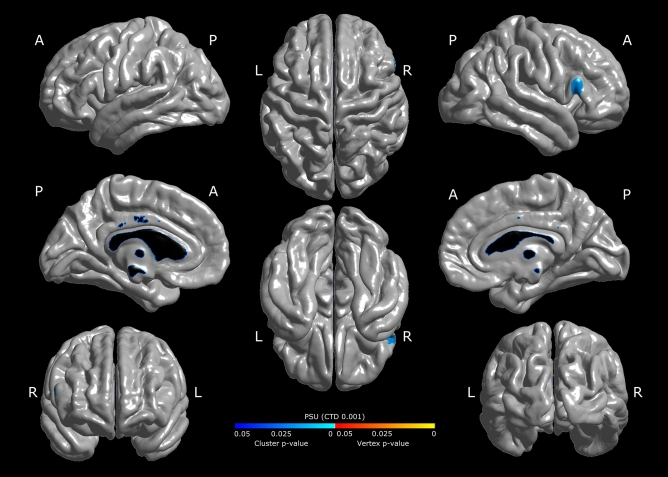
Significant clusters of the correlation between cortical thickness and the University Selection Test outcomes (PSU) (language + mathematics), corrected by sex and socioeconomic status. Color represents the *p*-value for a cluster in the right frontal gyrus that survives the multiple comparison correction (with the most demanding correction, CTD p < 0.001, cluster corrected p < 0.05). A: anterior; P: posterior, R: right, L: left.

We performed two analyses as follows. First, a whole-cortical analysis of cortical thinness was carried out using a general linear model with PSU outcomes (language + mathematics), sex, and SES as regressors. Then, a cortical and subcortical segmentation was carried out, including independent regions of interest (ROI) of cortical areas derived from the first analysis (see Methods). Thus, using cluster-level correction, the cortical thinness analyses showed that the right inferior frontal gyrus thickness (rIFG) positively correlated with PSU outcomes (CTD < 0.001, cluster corrected *p* < 0.05). Figure 2 shows the T-value of the correlation between cortical thickness and PSU outcomes (corrected by sex and SES). Figure 3 illustrates the p-value of this correlation (corrected by sex and SES) for clusters that survived the multiple comparison correction.

Next, cortical and subcortical brain segmentation was used to acquire relevant brain structural parameters. Volumes expressed as mean ± SD by sex and group are shown in Table 3. Males from the High SA Group had total gray matter (*p* = 0.0027), left cerebellum cortex (*p* = 0.0008), brainstem (*p* = 0.0023), left hippocampus (*p* = 0.0033), right cerebellum cortex (*p* = 0.0010), and right pallidum (*p* = 0.0051) volumes significantly higher than their peers from the Low SA Group, and the left accumbens area volume was significantly lower (*p* = 0.0098). Females from the High SA Group had brain segmentation without ventricles (*p* = 0.0017), total cortical gray matter (*p* = 0.0029), left hemisphere cortical gray matter (*p* = 0.0038), right hemisphere cortical gray matter (*p* = 0.0022), total gray matter (*p* = 0.0003), supratentorial (*p* = 0.0063), left cerebellum cortex (*p* = 0.0065), brain-stem (*p* = 0.0009), right cerebellum WM (*p* = 0.0084) and cortex (*p* = 0.0009) volumes significantly greater than their peers from the Low SA Group.

**Table 3:** Brain structural parameters volumes by sex and group.

Brain structural parameters volumes	Males			Females		
	High SA Group (n = 42)	Low SA Group (n = 16)	*p-*value	High SA Group (n = 21)	Low SA Group (n = 23)	*p-*value
	volumes (cc)					
Brain segmentation without ventricles	1279.36 ± 82.80	1229.29 ± 114.67	.0705	1165.78 ± 93.18	1083.98 ± 68.16	.0017**
Cortical gray matter	547.76 ± 41.52	525.91 ± 40.56	.0768	500.09 ± 37.74	465.76 ± 34.19	.0029**
Left hemisphere cortical gray matter	274.23 ± 20.95	261.82 ± 20.46	.0473*	249.45 ± 19.56	232.39 ± 17.43	.0038**
Right hemisphere cortical gray matter	273.54 ± 20.73	264.08 ± 20.28	.1242	250.64 ± 18.31	233.38 ± 16.88	.0022**
Cerebral white matter	496.90 ± 39.76	493.80 ± 64.88	.8257	453.56 ± 42.91	427.64 ± 39.09	.0421*
Left hemisphere cerebral white matter	248.80 ± 19.89	246.38 ± 32.45	.7309	226.85 ± 21.85	213.14 ± 19.39	.0330*
Right hemisphere cerebral white matter	248.10 ± 19.97	247.42 ± 32.55	.9236	226.71 ± 21.14	214.50 ± 19.80	.0545
Gray matter	751.55 ± 48.08	705.31 ± 55.26	.0027**	681.11 ± 52.12	628.74 ± 34.93	.0003***
Subcortical gray matter	66.38 ± 4.49	62.90 ± 5.76	.0181*	60.99 ± 4.89	57.68 ± 4.38	.0224*
Supratentorial	1130.84 ± 79.52	1101.80 ± 101.68	.2554	1032.82 ± 81.59	967.03 ± 70.09	.0063**
Left cerebellum white matter	15.86 ± 2.22	15.67 ± 2.28	.7690	16.08 ± 2.81	14.42 ± 1.77	.0235*
Left inferior frontal gyrus	2.71 ± 0.14	2.65 ± 0.16	.2202	2.76 ± 0.18	2.63 ± 0.12	.0172*
Left cerebellum cortex	65.54 ± 8.71	56.82 ± 7.34	.0008***	57.87 ± 8.31	51.76 ± 5.71	.0065**
Left thalamus proper	8.32 ± 0.69	8.02 ± 0.83	.1729	7.85 ± 0.94	7.30 ± 0.61	.0252*
Left caudate	3.99 ± 0.47	3.82 ± 0.49	.2233	3.84 ± 0.46	3.55 ± 0.41	.0363*
Left putamen	6.02 ± 0.61	5.77 ± 0.81	.2155	5.63 ± 0.69	5.50 ± 0.77	.5610
Left pallidum	2.21 ± 0.24	1.95 ± 0.45	.0081**	1.86 ± 0.34	1.68 ± 0.34	.0919
Brainstem	24.01 ± 3.01	21.19 ± 2.98	.0023**	21.65 ± 2.83	19.01 ± 1.79	.0006***
Left hippocampus	4.69 ± 0.38	4.31 ± 0.48	.0033**	4.33 ± 0.46	4.09 ± 0.45	.0823
Left amygdala	1.82 ± 0.15	1.85 ± 0.17	.5701	1.58 ± 0.15	1.57 ± 0.16	.8600
Left accumbens area	0.54 ± 0.12	0.64 ± 0.12	.0098**	0.56 ± 0.15	0.56 ± 0.11	.8324
Left ventral dorsal caudate	4,61 ± 0.41	4.40 ± 0.46	.0947	4.12 ± 0.36	3.86 ± 0.35	.0186*
Right inferior frontal gyrus	2.82 ± 0.20	2.66 ± 0.12	.0039**	2.84 ± 0.23	2.65 ± 0.18	.0036**
Right cerebellum white matter	15.26 ± 2.17	15.08 ± 2.26	.7798	15.59 ± 2.41	13.83 ± 1.79	.0084**
Right cerebellum cortex	67.20 ± 8.53	58.79 ± 7.50	.0010**	59.70 ± 7.83	52.29 ± 5.84	.0009***
Right thalamus proper	7.97 ± 0.64	7.62 ± 0.73	.0806	7.29 ± 0.69	6.87 ± 0.57	.0368*
Right caudate	4.04 ± 0.45	3.85 ± 0.48	.1535	3.91 ± 0.48	3.59 ± 0.40	.0227*
Right putamen	5.99 ± 0.58	5.77 ± 0.72	.2506	5.52 ± 0.52	5.37 ± 0.59	.3662
Right pallidum	2.16 ± 0.23	1.86 ± 0.36	.0003***	1.84 ± 0.35	1.70 ± 0.25	.1220
Right hippocampus	5.99 ± 0.58	4.60 ± 0.47	.0380*	4.50 ± 0.53	4.30 ± 0.43	.1632
Right amygdala	2.02 ± 0.19	1.96 ± 0.17	.2280	1.75 ± 0.18	1.63 ± 0.16	.0234*
Right accumbens area	0.70 ± 0.10	0.65 ± 0.09	.0732	0.64 ± 0.10	0.58 ± 0.07	.0259*
Right ventral dorsal caudate	4.59 ± 0.37	4.32 ± 0.43	.0213*	4.09 ± 0.36	3.83 ± 0.31	.0147*
Posterior corpus callosum	0.96 ± 0.15	1.02 ± 0.21	.2379	1.05 ± 0.13	0.96 ± 0.13	.0347*
Middle-posterior corpus callosum	0.49 ± 0.11	0.57 ± 0.12	.0192*	0.51 ± 0.12	0.52 ± 0.15	.8776
Central corpus callosum	0.53 ± 0.15	0.63 ± 0.17	.0414*	0.56 ± 0.14	0.52 ± 0.12	.2800
Middle-anterior corpus callosum	0.58 ± 0.18	0.63 ± 0.20	.3889	0.61 ± 0.16	0.58 ± 0.14	.5299
Anterior corpus callosum	0.90 ± 0.18	0.97 ± 0.19	.1957	0.97 ± 0.16	0.93 ± 0.17	.4793

Results are expressed as mean ± SD. Means were compared by Bonferroni's test. SA: scholastic achievement; PSU: University Selection Test; High SA Group: High PSU scores (> 620, > p75); Low SA Group: Low PSU scores (< 450, < p25). Bonferroni corrected p < .05*; < .01**; < .001***

**Figure 4:**
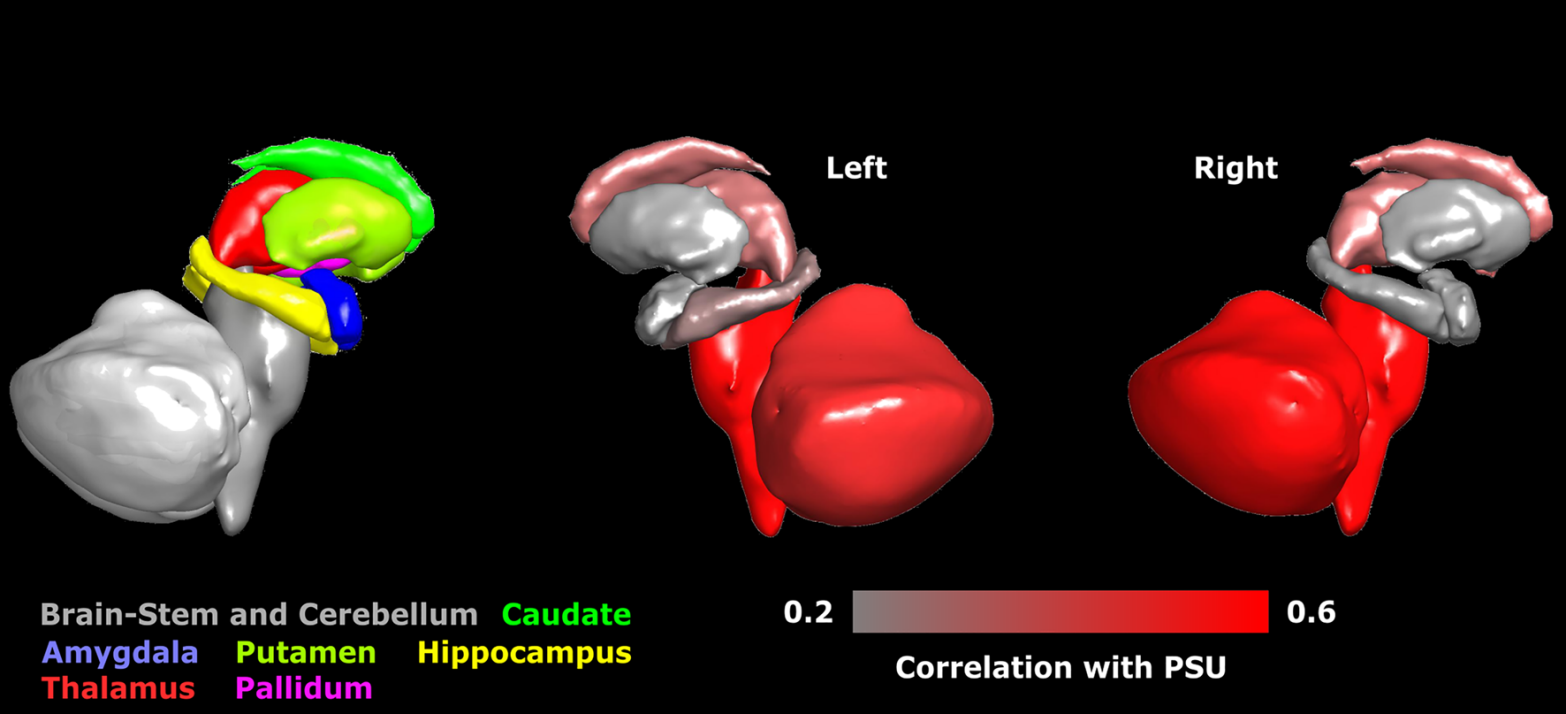
Left: Individual example of subcortical segmentation. Colors represent the different structures used in the correlation analysis (Brainstem and cerebellum, Caudate, Amygdala, Putamen, Hippocampus, Thalamus, and Pallidum). Right: Correlation between the volume of each structure and the University Selection Test (PSU) outcomes for both males and females. Color represents the correlation coefficient.

Finally, Pearson correlations between the brain structural volume parameters and the PSU outcomes were carried out by pooling High SA and Low SA groups by sex (see Methods and Table 4). High correlations were observed, especially in females, for brain segmentation without ventricles volume and outcomes in mathematics, GMV with language and mathematics PSU outcomes, and brain-stem and right cerebellum cortex with PSU mathematics outcome. Figure 4 shows the correlation analysis between subcortical volume and the PSU outcomes (language + mathematics) for both sexes.

**Table 4:** Pearson correlation coefficients between brain structural parameters volumes and the University Selection Test outcomes by sex.

Brain structural parameters volumes	PSU (L + M)		Language (L)		Mathematics (M)	
	Males	Females	Males	Females	Males	Females
Brain segmentation without ventricles	.302	.506**	.353*	.458*	.247	.512***
Cortical gray matter	.326*	.465*	.364*	.443*	.275	.449*
Left hemisphere cortical gray matter	.341*	.456*	.373*	.442*	.297*	.433**
Right hemisphere cortical gray matter	.308*	.471*	.352*	.442*	.250	.462**
Cerebral white matter	.088	.345*	.149	.301	.035	.361*
Left hemisphere cerebral white matter	.096	.361*	.153	.318*	.047	.373*
Right hemisphere cerebral white matter	.080	.328*	.144	.282	.022	.347*
Gray matter	.445*	.574***	.477*	.533***	.400*	.568***
Subcortical gray matter	.324*	.362*	.378**	.317*	.266*	.378*
Supratentorial	.227	.435*	.285	.401*	.166	.433**
Left inferior frontal gyrus	.177	.356*	.122	.383*	.153	.303
Left cerebellum white matter	.053	.323*	.119	.257	−.004	.361*
Left cerebellum cortex	.395*	.487*	.387*	.429*	.396*	.504**
Left thalamus proper	.224	.343*	.248	.315*	.193	.343*
Left caudate	.175	.350*	.230	.338*	.129	.333*
Left putamen	.190	.041	.264*	−.007	.094	.083
Left pallidum	.261	.357*	.257	.355*	.275	.331*
Brainstem	.357*	.575***	.383*	.478*	.321*	.623***
Left hippocampus	.433*	.299	.461*	.244	.375*	.329*
Left amygdala	−.037	−.001	−.039	−.038	−.033	.035
Left accumbens area	−.242	−.160	−.201	−.183	−.280	−.124
Left ventral dorsal caudate	.221	.382*	.276*	.320*	.180	.410**
Right inferior frontal gyrus	.346*	.540***	.330*	.443**	.345**	.590****
Right cerebellum white matter	.045	.372*	.116	.302*	−.013	.409**
Right cerebellum cortex	.390*	.565***	.386*	.496**	.386*	.585***
Right thalamus proper	.226	.349*	.268	.347*	.192	.324*
Right caudate	.206	.363*	.258	.345*	.162	.353*
Right putamen	.158	.083	.218	.041	.010	.116
Right pallidum	.414*	.354*	.415*	.340*	.405*	.340*
Right hippocampus	.320*	.221	.366*	.151	.256	.271
Right amygdala	.178	.309*	.201	.304*	.161	.290
Right accumbens area	.302	.370*	.360*	.306*	.214	.402*
Right ventral dorsal caudate	.303*	.431*	.348*	.393*	.270	.434**
Posterior corpus callosum	−.227	.318*	−.208	.346*	−.227	.265
Middle-posterior corpus callosum	−.327*	−.082	−.306*	−.088	−.308*	−.069
Central corpus callosum	−.242	.116	−.229	.124	−.238	.099
Middle-anterior corpus callosum	−.140	.045	−.142	−.013	−.113	.096
Anterior corpus callosum	−.239	.067	−.228	.089	−.226	.040

PSU: University Selection Test; FDR q < .05 *; q < .01**; q < .001 ***.

### Pearson correlation coefficients matrix between PSU scores and most significant parameters

Pearson's canonical and partial correlations were conducted to assess which significant areas in the initial findings better explain the SA variance (see Table 5). For the rIFG, the main result of the whole-cortical analysis, the volume was extracted using an independent ROI (see Methods section). Regarding partial correlations (Table 5B), two independent correlations were analyzed: the first includes total PSU scores, and the second includes language and mathematics scores separately. In these analyses, positive and significant correlations were observed between IA and PSU scores for language (*p* < 0.0001) and mathematics (*p* < 0.0001). The PSU outcomes positively and significantly correlated with IA (*p* < 0.0001), GMV (*p* = 0.0022) and rIFG (*p* = 0.0140). Language scores were positively and significantly correlated with GMV (*p* = 0.0430), and mathematics scores with IA (*p* < 0.0001) and rIFG (*p* = 0.0110). In addition, total GMV was positively and significantly correlated with Z-HC (*p* < 0.0001) and negatively correlated with BMI (*p* < 0.0001).

**Table 5:** Pearson correlation coefficients (A) and partial correlation coefficients (B) matrices between the University Selection Tests scores and most significant parameters*.*

	PSU	LPSU	MPSU	IA	GMV	rIFG	BW	BL	Z-HC	BMI
**(A)**										
LPSU	.959****	–								
MPSU	.969****	.860****	–							
IA	.805****	.758****	.794****	–						
GMV	.575****	.563****	.548****	.460****	–					
rIFG	.442****	.395****	.456****	.392****	.264**	–				
BW	.139	.139	.127	.108	.228*	.029	–			
BL	.252*	.249*	.233*	.182	.154	.007	.654****	–		
Z-HC	.393****	.407****	.335***	.294**	.717****	.107	.316**	.258*	–	
BMI	−.261**	−.217*	−.293**	−.275**	−.114	.113	.224*	−.016	.333***	–
SES	.561****	.567****	.516****	.440****	.361****	.187	.056	.142	.317**	−.094

	LPSU	MPSU	PSU	IA	GMV	BW	BL	Z-HC	BMI	SES
**(B)**										
MPSU	.23**									
IA	.420***	.470***	.670***							
GMV	.210*	.160	.310**	−.090						
BW	−.110	−.080	−.090	.100	.130					
BL	.130	.040	.170	−.120	−.150	.560***				
Z-HC	.005	−.020	−.030	.080	.670***	.070	.050			
BMI	.100	.030	.060	−.230*	−.390***	.190	−.160	.510***		
SES	.140	.140	.280**	.020	.007	.060	.040	.060	.010	
rIFG	.070	.260*	.240*	−.040	.120	−.190	.120	−.080	.110	.030

PSU: University Selection Test; LPSU, language University Selection Test score; MPSU, mathematics University Selection Test score; IA, intellectual ability; GMV, gray matter volume; BW, birth weight; BL, birth length; Z-HC, head circumference-for-age Z-score; BMI, body mass index; rIFG, right inferior frontal gyrus thickness. * *p* < .05; ** *p* < .01; *** *p* < .001; **** *p* < .0001.

### Multiple regression analysis between PSU outcomes (dependent variable) and most relevant parameters (independent variables)

The multiple regression analysis revealed that, independently of sex, IA, GMV, rIFG, and SES were the independent variables more significantly associated with PSU outcomes *(R*^2^ = 0.811, Table 6). The same was observed for the mathematics score (*R*^2^ = 0.750) and the language score (*R*^2^ = 0.770), except for the rIFG for the latter, which was not significant.

**Table 6:** Multiple regression analysis between the University Selection Test (PSU) (mean language + mathematics), language PSU and mathematics PSU scores (dependent variables) and the most relevant parameters (independent variables).

Parameter	Estimate	Standard Error of Estimate	T for H0: Parameter = 0	*p* >|T|
**PSU score (mean language + mathematics)**				
Intercept	−291.5	139.4	−2.09	.0393
**IA (Ref: Grades IV + V)**				
Grade I + II	239.3	26.9	8.87	.0001
Grade III	49.8	26.0	1.92	.0586
GMV	0.59	0.15	3.90	.0002
rIFG	106.9	37.4	2.86	.0053
**SES (Ref: Medium)**				
High SES	16.6	20.1	0.83	.4097
Low SES	44.6	18.4	2.41	.0178
**Sex (Ref: males)**				
Females	−27.8	19.1	1.45	.1505
**Language PSU score**				
Intercept	−251.3	148.0	−1.70	.0930
**IA (Ref: Grades IV + V)**				
Grade I + II	235.9	28.6	8.24	.0001
Grade III	68.7	27.6	2.49	.0146
GMV	0.64	0.16	4.03	.0001
**SES (Ref: Medium)**				
High SES	26.9	21.3	1.26	.2113
Low SES	−41.5	19.6	−2.12	.0368
rIFG	71.0	39.7	1.79	.0770
**Sex (Ref: males)**				
Females	−35.8	20.3	1.76	.0817
**Mathematics PSU score**				
Intercept	−321.8	175.5	−1.83	.0699
**IA (Ref: Grades IV + V)**				
Grade I + II	238.8	32.8	7.27	.0001
Grade III	27.5	32.0	0.86	.3912
rIFG	142.9	47.5	3.00	.0034
GMV	0.52	0.19	2.75	.0071
**SES (Ref: Medium)**				
High SES	6.08	25.5	0.24	.8127
Low SES	−49.1	23.2	−2.12	.0370
**Sex (Ref: males)**				
Females	−18.3	24.1	0.76	.4494

Model *R*^2^ = .811; Root MSE (Root mean squared error, standard deviation of the dependent variable PSU) = 71.4949; Model *F* Value = 55.76, *p* < .0001.

Model *R*^2^ = .770; Root MSE (Root mean squared error, standard deviation of the dependent variable language PSU) = 75.9123; Model *F* Value = 43.61, *p* < .0001.

Model *R*^2^ = .750; Root MSE (Root mean squared error, standard deviation of the dependent variable mathematics PSU) = 90.9008; Model *F* Value = 39.52, *p* < .0001.

IA, intellectual ability; IA grades: Grade I, superior; Grade II, above average; Grade III, average; Grade IV, below average; Grade V, intellectually defective. GMV, gray matter volume. rIFG, right inferior frontal gyrus thickness. SES, socio-economic status. The initial regressors (independent variables considered in the statistical model) considered for the forward stepwise selection method were IA, SES, sex, brain segmentation without ventricles, GMV, brainstem, rIFG, right cerebellum cortex, left cerebellum cortex.

## Discussion

The present results support the study hypothesis, revealing that independently of sex, IA, GMV, rIFG, and SES were the variables more significantly associated with PSU outcomes. The total variability observed in the PSU scores is explained as 81.1% *(R*^2^ = 0.811) by the effect of these variables. The results also reveal that the total GMV and thickness of the rIFG explain the SA variance independently of the IA. These findings were observed in both the language and mathematics scores, except for the rIFG in the language outcomes.

Several findings have displayed that IA is the most stable and powerful predictor of SA in standardized tests^1,2,13,14,17–19,56–64^. The mean correlation between general intelligence and academic performance is approximately 0.50, but it varies considerably depending on the variability of the measures and samples^14,65–67^. In our study, the correlation between IA and PSU scores was 0.67, which agrees with our previous findings in high school graduates^19^. Interestingly, our findings indicated that brain measures correlated with SA independently of IA, suggesting that this marker could be more specifically associated with SA.

The multiple regression analysis of brain structural parameters associated with PSU outcomes showed that GMV and the rIFG were the most relevant brain parameters. In the present study, a high correlation was found between GMV and Z-HC, a physical marker of past nutrition and brain development and an important anthropometric indicator associated with SA and IA consistently reported in the literature^24,27–30,68,69^. Even though the males of the High SA Group exhibited higher values of Z-HC than the rest of the sample, the results presented here suggest that Z-HC may be a significant indicator of IA or SA only for females. Findings by several authors have shown that total brain volume is a good predictor of IA, specially GMV is associated with higher IA^70,71^. These findings have been interpreted as the general intelligence depends on distributed areas throughout the brain^72–74^.

Despite the plentiful research investigating the relationship between brain structure and intelligence, few studies have focused on the relationship between the brain and SA. Prior work shows that prefrontal GM density correlated with SA, and this correlation is partially mediated by general intelligence^75^. Moreover, the association between frontal GM and SA persisted even after adjusting for family SES and IA^75^. The prefrontal cortex is commonly highlighted as the center of individual differences in general intelligence^76,77^. Baseline measurements of frontal GMV predict verbal episodic memory performance changes over ten years of follow-ups^78^. In the context of the results presented here, it is possible to postulate that frontal GM volumes could be a neuroanatomical marker for SA partially independent of IA.

Particularly for prefrontal regions, the current results show that the rIFG contributes to explaining PSU outcomes, but only in mathematics, which was unexpected. However, this result is in line with recent studies investigating this issue by measuring neural activity associated with numerical magnitude processing acuity, domain-general attention, and selective attention to numbers via functional MRI. Results showed that activation in the IFG predicted achievements in mathematics^79,80^. In children and adolescents, the resting-state analysis also reveals the association between IFG connectivity with intelligence^81^. Interestingly, the cerebellum cortex and the brainstem present a high correlation with PSU scores, although these parameters were not selected by the linear regression model. The cerebellum has been related to high cognitive function^82,83^ and likely presents an important role in SA that must be studied in further research. Similarly, several key neuromodulator systems that influence cognitive performance, such as locus coeruleus, are settled into the brainstem^84,85^. Studies with a greater spatial resolution are required to better identify the influence of these systems on SA.

Notable, the described brain-SA association was carried out within the framework of a multidimensional approach considering socioeconomic, intellectual, nutritional, and demographic variables. This approach is not only to control for these variables but also to understand SA as a complex social and biological phenomenon. Consequently, SA is associated with SES, maternal schooling, intelligence, and antecedents of malnutrition in the first year of life^70,86,87^. Accordingly, SES in our study was also significantly correlated with PSU outcomes, likely because poverty conditions are also associated with structural differences in several areas of the brain^86^. Other findings revealed that childhood SES predicts executive function performance and measures of prefrontal cortical function, specifically in the association between family income and parental education and GM thickness^86^.

Despite socioeconomic indicators, such as parental schooling, occupation of the household head, and housing characteristics, which were positive and significantly associated with PSU scores in both language and mathematics, SES was the only socioeconomic variable most significantly associated with PSU outcomes in the statistical regression model. Note that SES is a global construct that includes, among other indicators, parental education. In this context, several studies have emphasized that parental education is another relevant factor influencing brain development and SA. Parental IA (especially maternal IA) is consistent in explaining children’s IA, probably, because mothers are the primary source of intellectual stimulation and enrichment in the psycho-social environment and the health-related behavior of the family^88–91^. Parental education predicted cortical thickness in the right anterior cingulate gyrus and the IFG, providing a meaningful link between SES and cognitive function among healthy children^92^.

Our study has several limitations that must be considered when interpreting the presented results. First, the use of multiple brain parameters increases the type I error, although standard corrections for multiple comparisons were performed. Second, many variables such as breastfeeding, birth weight according to gestational age, Z-HC at birth, parental intelligence, and maternal stimulation at an early age could not be considered in the present analysis. Many of these variables were not registered in the hospital records, and the mothers did not remember them. Nor was it possible to measure the degree of parental stimulation because of the sample's age. Many parents were separated, unlocatable, or had died, so it was impossible to measure their intelligence with any reliability. In this regard, it has been found that breastfed children had significantly higher IA scores and larger brain volume, GMV, total cortical GM, and subcortical GMV compared with non-breastfed children^93^. Early postnatal nutrition is essential for brain growth and maturation, and WM connectivity strength may be a valuable predictor of long-term cognitive functioning^32,33,94^. In addition, it has been found that low-risk preterm children achieve lower scores in neurophysiological tests than children born at term, impacting brain volumes and cognitive outcomes in the long term^95–98^, although our study did not consider that variable. Another relevant issue is the possible differences in the incidence of developmental disorders between the two studied groups. Although participants have no history of or current developmental diagnosis, it is impossible to rule out undiagnosed conditions. The participants of this study were a group of high school graduates with a narrow age range. Future research should consider a wide range of factors, including elementary and high school students. Considering that SA consists of different complex abilities, future studies should focus on exploring the associations between SA and brain networks using task-based functional MRI. Therefore, more research is needed to elucidate and understand these mechanisms further.

Altogether, our findings present evidence that GMV and the rIFG serve as the neural basis of academic performance and reveal the role of general intelligence and SES in the association between brain structure and SA. Knowing the neuronal subtract of SA can improve a not well-known field of knowledge, shedding light on the possible cognitive mechanisms. Thus, the results are relevant in explaining the complex interactions among variables that affect PSU outcomes and can be helpful in the design and implementation of health and educational policies to improve scholar performance. PSU outcomes are crucial for students to pursue successful collegiate careers and to guide their future lives and prospects as adults by developing their talents and learning specific skills for desired careers. In this context, evidence-based public policies and interventions may help the most disadvantaged children through comprehensive health care, maternal education, and in-school care, enabling them to develop their talents and achieve their promises and goals.

## Data Availability

The datasets generated are available upon request from the corresponding author.
